# Lignosulfonate-Assisted In Situ Deposition of Palladium Nanoparticles on Carbon Nanotubes for the Electrocatalytic Sensing of Hydrazine

**DOI:** 10.3390/molecules28207076

**Published:** 2023-10-13

**Authors:** Patrycja Płócienniczak-Bywalska, Tomasz Rębiś, Amanda Leda, Grzegorz Milczarek

**Affiliations:** 1 Faculty of Chemistry, Adam Mickiewicz University, Uniwersytetu Poznańskiego 8, 61-614 Poznań, Poland; patrycja.plocienniczak@amu.edu.pl; 2 Institute of Chemistry and Technical Electrochemistry, Poznan University of Technology, Berdychowo 4, 60-965 Poznań, Poland; amanda.leda@doctorate.put.poznan.pl

**Keywords:** palladium nanoparticles, hydrazine, multi-walled carbon nanotubes, lignosulfonate, electrocatalysis, in situ

## Abstract

This paper presents a novel modified electrode for an amperometric hydrazine sensor based on multi-walled carbon nanotubes (MWCNTs) modified with lignosulfonate (LS) and decorated with palladium nanoparticles (NPds). The MWCNT/LS/NPd hybrid was characterized by atomic force microscopy (AFM), X-ray photoelectron spectroscopy (XPS), and X-ray diffraction (XRD). The electrochemical properties of the electrode material were evaluated using cyclic voltammetry and chronoamperometry. The results showed that GC/MWCNT/LS/NPd possesses potent electrocatalytic properties towards the electro-oxidation of hydrazine. The electrode demonstrated exceptional electrocatalytic activity coupled with a considerable sensitivity of 0.166 μA μM^−1^ cm^−2^. The response was linear from 3.0 to 100 µM L^−1^ and 100 to 10,000 µM L^−1^, and the LOD was quantified to 0.80 µM L^−1^. The efficacy of the modified electrode as an electrochemical sensor was corroborated in a study of hydrazine determination in water samples.

## 1. Introduction

Hydrazine (N_2_H_4_) is an important chemical compound, extremely reactive and strongly reducing. It is widely used in fuel cells, rocket fuel, antioxidant production, pesticide, herbicide, and insecticide synthesis, pharmaceutical applications, and the photographic industry [1,2,3,4,5,6,7]. As a result of its widespread and commercial use, hydrazine and its derivatives are often found in our environment, which can pose a serious threat to living organisms. Hydrazine has been identified as highly neurotoxic, carcinogenic, and mutagenic. The molecule can cause harmful effects. It damages the lungs, kidneys, liver, brain, spinal cord, and DNA [8,9,10,11,12]. Concrete steps to detect hydrazine in our environment are needed because of its increasing consumption. The development of simple and sensitive analytical techniques to deal with high concentrations of hydrazine in our environment is becoming an important focus of research. There are several effective methods for the determination of traces of hydrazine. These include chemiluminescence [13], spectrometry [14,15,16,17], chromatography [18,19,20], and modified electrodes [21,22,23,24]. Electrochemical analysis appears to be a promising alternative due to its simplicity and low cost, while maintaining high activity and sensitivity. The development of high-performance electrocatalysts for the detection and/or electro-oxidation of hydrazine is still being pursued by researchers.

Using electrodes modified with various metal nanoparticles or metal oxide nanoparticles can resolve this issue. Nanostructures demonstrate outstanding performance due to their morphology, including a vast surface area that enhances electron transfer efficiency and the availability of active sites to interact with more molecules during the oxidation process. Nanostructures composed of either metals or metal oxides must be chosen based on their specific application requirements, considering factors such as their composition, shape, size, and cost [25,26]. Particularly noteworthy are noble metal nanoparticles (NMNPs), such as Pt, Ag, Au, and Pd. The unique features of NMNPs offer considerable electrocatalytic activity, improved mass transport, and increased surface area for the developed electrodes [27,28,29,30,31,32,33,34,35]. For the electrochemical detection of N_2_H_4_, palladium-based electrodes appear to be the most promising. The presence of palladium nanoparticles (NPds) on the electrode surface reduces the oxidation potential of hydrazine and leads to the formation of dinitrogen [36]. However, electrode modification with NPds alone does not guarantee the best possible results as the particles tend to aggregate into larger structures, resulting in reduced catalytic activity and instability [37]. Much better results are obtained by dispersing the nanoparticles in some type of matrix. Examples include graphene [38,39,40], metal oxides [41], carbon nanotubes [42,43,44,45], and polymers [46,47,48]. For instance, Promsuwan and colleagues developed an amperometric hydrazine sensor by modifying a glassy carbon electrode with palladium-poly(3,4-ethylene dioxythiophene) coated on carbon microspheres/graphene nanoplatelets (Pd-PEDOT@CM/GNP/GCE) [49]. The sensor exhibited a linear response within the range of 1.0 to 100 μM L^−1^ and from 100 to 5000 μM L^−1^, with a detection limit of 0.28 ± 0.02 μM L^−1^ and a sensitivity of 0.200 μA μM^−1^ cm^−2^. Ghasemi et al. put forth an amperometric nanosensor that featured platinum–palladium nanoparticles decorated with reduced graphene oxides. The sensor detected hydrazine within a linear response range of 0.007 to 5.5 mM and had a detection limit of 1.7 μM [50].

We chose softwood lignosulfonate (LS) as the matrix for the synthesis of dispersed palladium nanoparticles. The use of lignin, a naturally abundant by-product of the paper industry, offers an environmentally friendly alternative to artificial polymers. To date, many reports have been published on the synthesis of noble metal nanoparticles in a lignin matrix and their use in catalytic reactions. For example, Coccia et al. used lignin as a reducing and stabilising agent for a one-pot green method to obtain catalytically active platinum (Pt) and palladium (Pd) nanoparticles [51,52]. However, the synthesis proposed by these researchers required an elevated temperature, i.e., 80 °C. A different approach was proposed by Mohazzab et al., who modified a commercial calcium lignosulfonate with NPds using the laser ablation in liquid (LAL) technique [53]. The resulting composite was found to be effective for hydrogen storage in alkaline environments as well as for the environmental remediation of common contaminants in aqueous environments. Despite the use of room temperature and the absence of additional solvents or reducing agents, the approach involves the use of high-power lasers.

In the production of novel functional materials, there is a growing interest in adhering to the principles of Green Chemistry by reducing pollutants, preventing or minimising waste, utilising safer or non-toxic solvents, and relying on renewable resources. Environmentally friendly methods for synthesizing metals and metal oxides are being proposed more frequently [54,55]. Also, in prior research, we have introduced a sustainable method for the synthesis of silver and platinum nanoparticles on multi-walled carbon nanotubes supported by lignosulfonate. This approach omitted the need for special equipment or elevated temperatures, and the lignosulfonate functioned as both a reducing and stabilising agent [56,57]. By utilizing the interactions between LS methoxyphenol groups and noble metal ions, we were able to acquire catalytically active metal particles within the MWCNT/LS matrix. In this study, we suggest an equally straightforward and eco-friendly technique for nanoparticle preparation. However, we assume different interactions between palladium ions and lignosulfonate deposited on MWCNTs. LS/MWCNT has the potential to serve as a chelating agent for palladium ions. Furthermore, it is possible to produce NPds through the electrochemical reduction of adsorbed palladium ions on a multifunctional electrode surface. Enhanced electrocatalysis of hydrazine can be achieved by the synergistic cooperation of LS-coated MWCNTs with electrodeposited NPds. The proposed hybrid material exhibited strong electrocatalytic properties for hydrazine oxidation. To the best of the authors’ knowledge, this is the first report on the application of lignosulfonate/palladium-nanoparticle-based materials for hydrazine electrocatalysis.

## 2. Results and Discussion

### 2.1. Coordination of Palladium on MWCNT/LS and Electrochemical Formation of MWCNT/LS/NPd

**Scheme 1:**
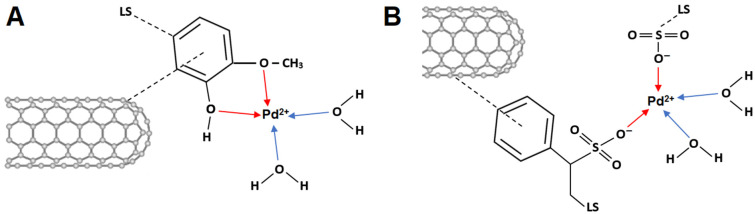
Schematic representation of potential MWCNT/LS-Pd^2+^ complex structures. Possible interactions between Pd^2+^ and methoxy groups (**A**). The complexation of Pd^2+^ by sulfonic groups of lignosulfonate (**B**).

The coordination bonds between transition metal ions and o-quinone molecules have already been demonstrated [58,59,60,61]. Consequently, LS can act as a potential matrix for the pre-concentration of metal cations due to its multifunctional structure, containing various functional groups (phenolic, quinone, carboxyl, sulfonic, and hydroxyl). Furthermore, chemical interactions can be established between LS and Pd^2+^ ions, and the possible interaction mechanism is presented in Scheme 1. When the MWCNT/LS hybrid is mixed with the PdCl_2_ solution for the intended duration, coordination bonds are formed, and Pd^2+^ accumulates on the surface of the MWCNT/LS.

**Figure 1:**
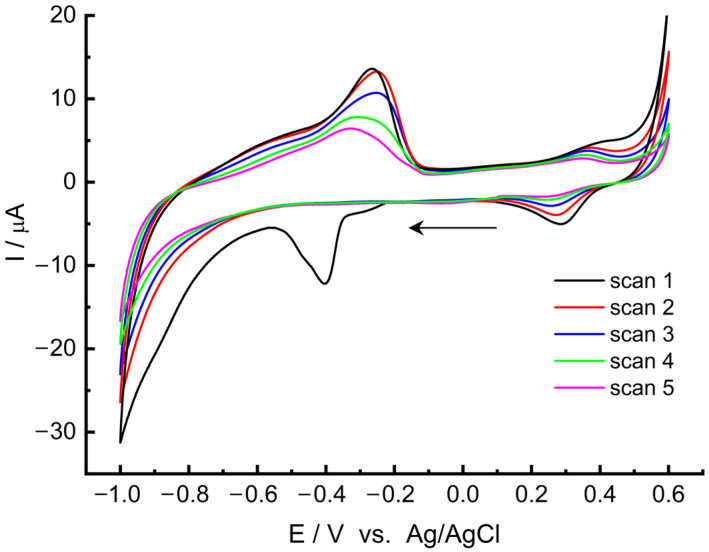
CV of GC/MWCNT/LS-Pd^2+^ recorded in 0.1 KCl. Scan rate: 10 mV s^−1^. The black arrow indicates the starting potential and the direction of the scan.

After immobilising Pd^2+^, the resulting MWCNT/LS-Pd^2+^ composite was deposited onto the GC surface. Afterwards, the modified electrode was transferred to a 0.1 KCl electrolyte (pH ~ 7) for CV measurements (Figure 1). In the initial scan, recorded from the open circuit potential (OCP) −0.21 V towards the negative potential, a well-defined cathode peak is visible at around −0.40 V. This peak is irreversible and vanishes in subsequent scans. The emergence of the peak can be attributed to the reduction of Pd^2+^ accumulated in the MWCNT/LS matrix, leading to the formation of palladium in a metallic form (Equation (1)). Prior studies by Velmurugan et al. [61,62] and Wen et al. [63] also acknowledged a comparable reduction peak of Pd^2+^.

Pd^2+^ + 2e^−^ → Pd^0^ (1)

Further scans reveal a standard electrochemical behaviour of metallic palladium. Hydrogen sorption signals and its accumulation processes were detected in the negative potential range (spanning from −0.6 V to −1.0 V and from −1.0 V to −0.1 V), which are specific to metallic palladium [36,63]. Furthermore, redox couples gradually disappear with an anodic peak at around 0.35 V and a cathodic peak at around 0.27 V, indicating the formation of palladium oxides and their subsequent reduction [36,63,64]. During the first five scans, the redox signal current decreases, indicating the gradual stabilization of NPds’ oxidation and reduction processes on the electrode surface. After five cycles, the electrode becomes stable and is fully prepared for further tests. Therefore, the electrode should be scanned five times before each measurement to properly prepare the electrode material for the experiments.

**Figure 2:**
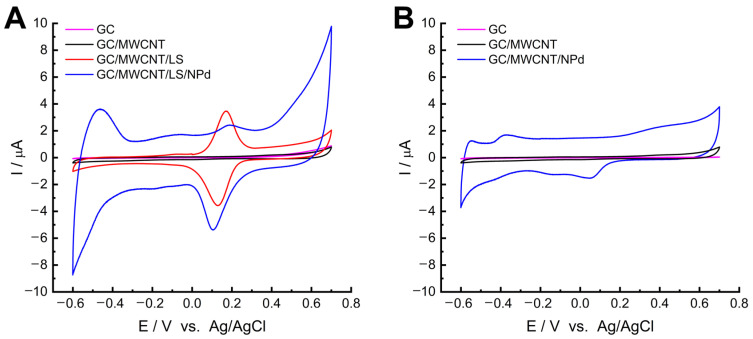
Cyclic voltammograms registered in 0.05 M PB buffer for (**A**) GC/MWCNT/LS/NPd and (**B**) GC/MWCNT/NPd. Scan rate: 10 mV s^−1^.

To verify that the LS can improve the accumulation of Pd^2+^, we performed a control test for the GC electrode modified by MWCNTs with adsorbed Pd^2+^ (GC/MWCNT-Pd^2+^). The CVs of GC/MWCNT/LS/NPd and GC/MWCNT/NPd recorded in 0.05 M PB buffer (pH = 7.4) are presented in Figure 2. Integrating the charge under the redox peaks allows the assessment of the palladium accumulation. The findings indicate that NPds may be deposited on the surface of the pristine MWCNTs, most likely due to the numerous oxygen-containing functional groups present in the structure [65,66]. Nonetheless, notably greater peak currents are evident on GC/MWCNT/LS/NPd as opposed to GC/MWCNT/NPd. This outcome can be accredited to the augmented quantity of chelated metal ions. It suggests that the adsorbed LS can enhance the chelating efficiency of MWCNTs to Pd^2+^ [65,66].

**Figure 3:**
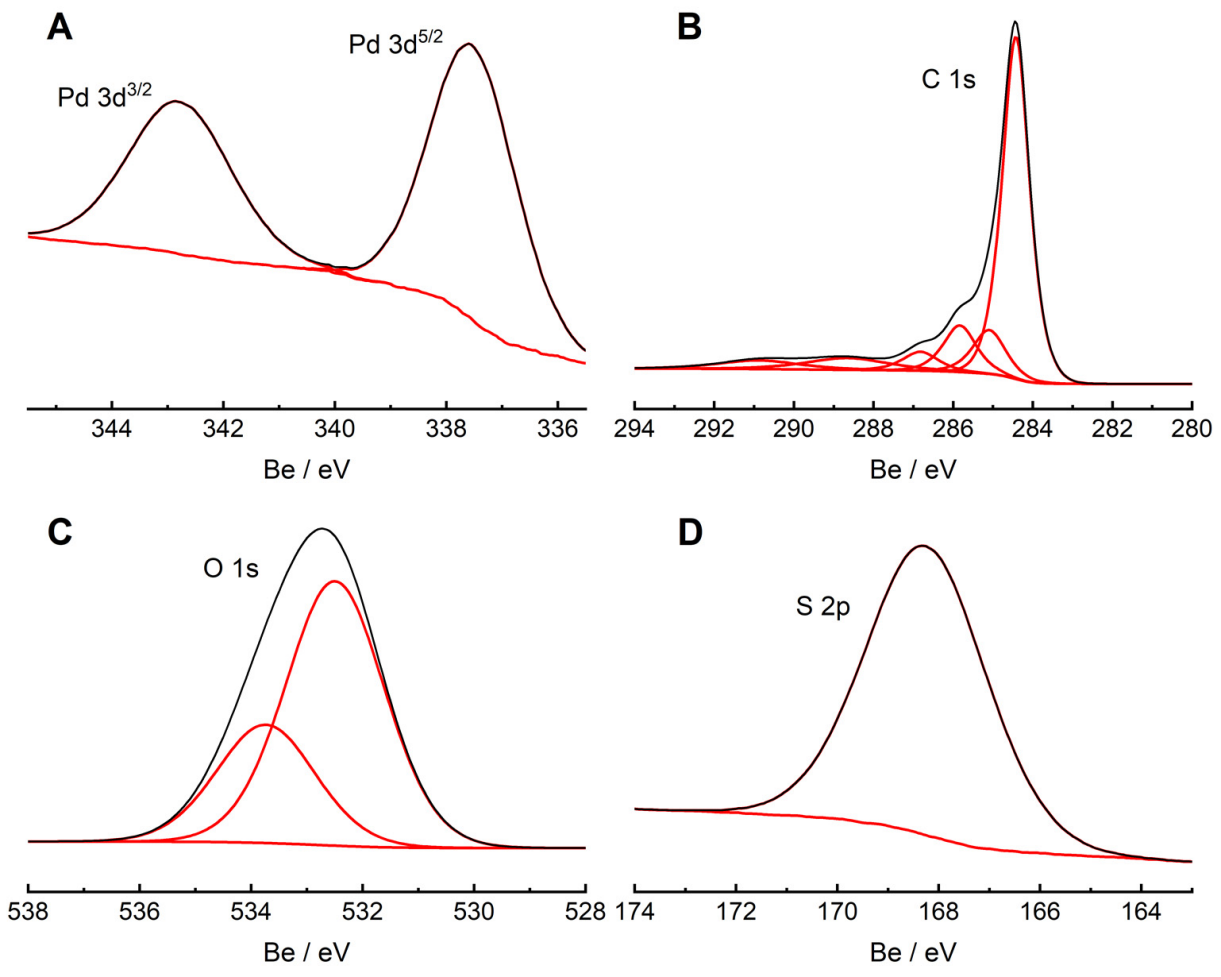
XPS spectrum recorded for MWCNT/LS/NPd hybrid material: (**A**) Pd 3d, (**B**) C 1s, (**C**) O 1s, and (**D**) S 2p.

To verify the existence of palladium ions accumulated within the MWCNT/LS matrix, the XPS technique was also applied. Figure 3A shows the high-resolution 3d Pd spectrum of the MWCNT/LS-Pd^2+^ hybrid material. In this spectrum, a Pd 3d 3/2 and 3d 5/2 doublet was recorded at the binding energies of 342.9 eV and 337.6 eV, respectively, and shifted by 2.3 eV in comparison to the palladium metal position (335.3 eV) [67,68]. The peak pair is quite narrow; the full width at half maximum is 1.9 eV. Both the position of these two peaks and their characteristics indicate the presence of palladium in the formal oxidation state +2 and determine the formation of a palladium complex in the sample [69,70]. Figure 3B shows a C 1s spectrum, and the major peak at about 284.5 eV can be attributed to the sp^2^-hybridized carbon atoms present in the MWCNT backbone [71]. A peak at 285.2 eV relates to sp^3^ C–C bonds, typical for MWCNTs. The peaks at 286.4, 287.7, 289.4, and 291.0 eV correspond to C–O bonds, C=O carbonyl groups, O–C=O carboxyl groups, and π–π*, respectively [71,72,73]. Figure 3C shows the deconvoluted peaks of O 1s at 532.5 eV and 533.9 eV, attributed to C=O and C–O–H groups, respectively. [56,57,74]. Moreover, a peak at 168.4 eV, which refers to S 2p, was recorded in the spectrum (Figure 3D). It indicates the presence of oxidized sulfur from the sulfonic group, typical for lignosulfonate [56,57].

### 2.2. Physicochemical Analysis of MWCNT/LS/NPd

**Figure 4:**
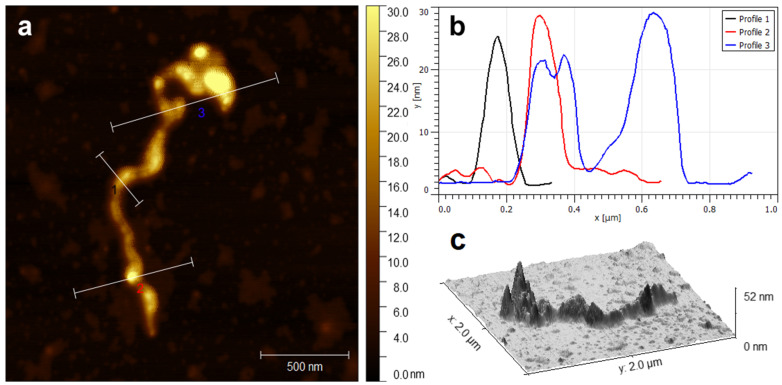
Physicochemical analysis of MWCNT/LS/NPd. (**a**) AFM image with corresponding cross-section locations, (**b**) AFM cross-section of MWCNT/LS/NPd, and (**c**) 3D visualization of MWCNT/LS/NPd.

The study of the morphology of palladium nanoparticles on the lignosulfonate-coated carbon nanotubes was performed using AFM microscopy. Figure 4 shows the AFM picture with cross-sections and 3D visualization for MWCNT/LS/NPd. The average value of the diameter of the developed material in the cross-section is about 70.69 ± 7.77 nm, which indicates good coverage of the MWCNT surface by LS and numerous Pd nanostructures. Compared to the average cross-sectional diameters of pure MWCNTs (39.25 ± 3.74 nm) and MWCNT/LS (48.20 ± 7.42 nm) presented in our previous work [57], it is concluded that the synthesis effectively resulted in a modified MWCNT/LS/NPd hybrid material with a differentiated surface area. Nanostructured palladium seems to be uniformly dispersed throughout the composite. Metallic objects in the form of spherical particles have been detected within the MWCNT/LS matrix. Moreover, the rough surface of the MWCNT/LS/NPd can be observed. The average roughness of the material is 2.62 ± 1.21 nm and is much higher than the average roughness of pristine MWCNTs (0.30 ± 0.07 nm) and MWCNT/LS (1.49 ± 0.60 nm). This is due to the presence of multiple palladium structures deposited on the MWCNT/LS in the reduction process.

The morphology and crystal structure of the MWCNT/LS/NPd hybrid material were examined using the XRD technique, as illustrated in Figure S1. The results conclusively demonstrate the presence of palladium in the sample, thereby affirming the successful deposition of its particles in the synthesis process. Notably, broad peaks were recorded at 2 theta around 40.1° and 46.5°. The positions correspond to the grid planes (111) and (200), respectively, and are correlated with the JCPDS card 05-0681 [75,76]. Furthermore, the peak shapes suggest that the immobilized palladium species have a non-crystalline, presumably amorphous structure. In addition, the signals present in the diffractogram at 2 theta around 26.0° and 42.6° are from MWCNTs, corresponding to reflections (002) and (100) typical of graphite structures (JCPDS card 01-0646) [77,78].

### 2.3. Electrochemical Study of GC/MWCNT/LS/NPd

**Figure 5:**
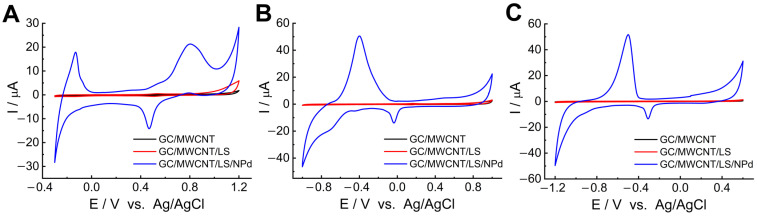
CVs of GC/MWCNT/LS/NPd recorded in (**A**) 0.1 M HClO_4_, (**B**) 0.05 M PB buffer, and (**C**) 0.1 M NaOH. Scan rate: 10 mV s^−1^.

The electrochemical study of the MWCNT/LS/NPd material was performed at the GC electrode using the CV technique. To best investigate the electrochemical performance of the modified electrode, measurements were carried out in three different electrolytes with various pH: 0.1 M HClO_4_ (pH = 1.1), 0.05 M PB buffer (pH = 7.4), and 0.1 M NaOH (pH = 13.0). The recorded voltammograms are shown in Figure 5. Analysis of the CV responses reveals typical signatures for palladium-based electrodes, including characteristic peaks for hydrogen adsorption/desorption [79,80,81,82,83,84,85]. Specific palladium redox processes also occurred, which were particularly evident in 0.1 M HClO_4_ (Figure 5A), presumably due to the higher concentration of H^+^ ions in the electrolyte. A broad wave of palladium oxide film formation was recorded from 0.7 V to 1.0 V, followed by a peak of reverse cathodic reduction of palladium oxides at E^0′^ = 0.45 V.

The electrocatalytic activity of the modified electrode is anticipated to rely on the number, size, and surface area of NPds. Meanwhile, the properties of nanostructures can be linked to the quantity of LS adsorbed on the surface of a singular MWCNT. Consequently, we sought to compare the electrochemical properties of electrode materials with varying LS loadings to determine the most catalytically active electrode. A range of samples were prepared, with varying amounts of LS on the surface of the MWCNTs. A variety of LS concentrations were used, spanning from 0.4 to 5.0 mg mL^−1^. Meanwhile, the PdCl_2_ concentration remained constant at 0.01 M. The range of LS concentrations was limited between 0.4 and 5.0 mg mL^−1^ as concentrations below this range would not ensure adequate chemical interactions between LS and Pd^2+^ ions. Conversely, higher concentrations inevitably resulted in strong LS adsorption on the MWCNTs’ surface, leading to subsequent aggregation and thus impeding the access of analytes to the active nanoparticles [56,57].

To verify this phenomenon, an additional experiment was carried out to determine the amount of LS adsorbed on the MWCNTs’ surface as a function of the LS concentration used in the synthesis. The concentration range for LS was 0.1–20.0 mg mL^−1^. The MWCNT/LS hybrid materials obtained during our study were analysed in relation to the current generated by the quinone/hydroquinone redox pair (Figure S2). The current dependence on LS concentration has a similar shape to the Langmuir adsorption isotherm. The current values rapidly increase for samples containing 0.1 to 1.0 mg mL^−1^, then gradually plateau at higher LS concentrations. Thus, the adsorption process can be considered as controlled.

**Figure 6:**
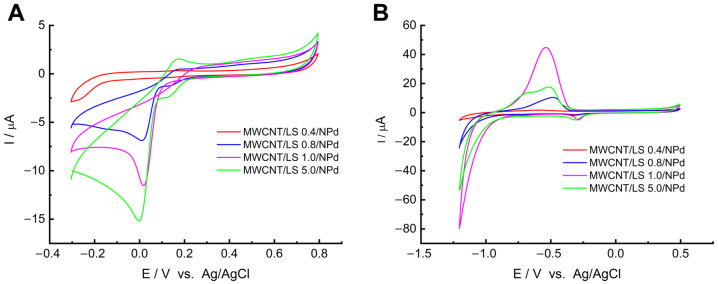
(**A**) Cyclic voltammograms of GC/MWCNT/LS/NPd electrodes obtained with different LS concentrations (from 0.4 to 5.0 mg mL^−1^). CVs recorded in PB buffer (0.05 M). Scan rate: 10 mV s^−1^. (**B**) Cyclic voltammograms recorded in NaOH (0.1M) for different GC/MWCNT/LS/NPd electrodes. Scan rate: 10 mV s^−1^.

The effect of LS concentration on electrochemical activity was assessed with CV in PB buffer (0.05 M) in the presence of oxygen (Figure 6A). The comparison of electrode materials can be achieved by contrasting their oxygen reduction capabilities. As depicted in Figure 6A, electrodes with LS concentrations of 0.8 mg mL^−1^ or more recorded an irreversible cathodic oxygen reduction peak at 0.0 V. With increasing quantities of LS, an increase in cathode current was noted at 0.0 V. However, the relationship between the quantity of nanoparticles in the electrode material and the quantity of LS present is non-linear (according to the quasi-Langmuir adsorption isotherm in Figure S2). There is a noticeable correlation between the number of nanoparticles and the level of LS loading in the electrode material. The oxygen reduction peak from the current experiment is consistent with these findings, indicating the presence of both LS and NPds. The more NPds on the MWCNT/LS surface, the more catalytically active sites and, thus, higher oxygen reduction currents.

In order to definitively determine the optimum LS concentration, the electrodes were tested for hydrogen evolution. The hydrogen sorption capacity was investigated in a solution of 0.1 M NaOH, which we purged of dissolved oxygen with nitrogen for a few minutes before the experiment (Figure 6B). Throughout the measurements, we maintained the system in a nitrogen atmosphere. The cyclic voltammetry (CV) response indicates that, as the concentration of LS increases, the overpotential for hydrogen evolution generally becomes less negative. Furthermore, a swift increase in the cathodic current occurred at −1.2 V, which is connected to processes of hydrogen sorption. This occurrence was particularly noticeable for the material with an LS loading of 1.0 mg mL^−1^, and the current value recorded was approximately −79 µA. Furthermore, the signal for anodic hydrogen accumulation was highest at −0.52 V for the electrode with an LS loading of 1.0 mg mL^−1^. Consequently, MWCNT/LS/NPd with an LS concentration of 1.0 mg mL^−1^ was chosen as the electrode material that is most catalytically active.

### 2.4. Electrocatalytic Hydrazine Oxidation at GC/MWCNT/LS/NPd

**Figure 7:**
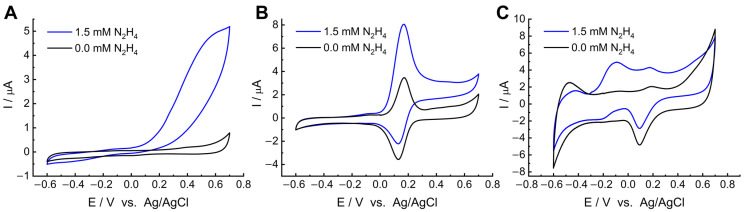
CVs of (**A**) GC/MWCNT, (**B**) GC/MWCNT/LS, and (**C**) GC/MWCNT/LS/NPd recorded in 0.05 M PB buffer in the absence and presence of 1.5 mM N_2_H_4_. Scan rate: 10 mV s^−1^.

The electrocatalytic activity of MWCNT/LS/NPd towards hydrazine oxidation was first assessed with cyclic voltammetry in 0.05 M PB with and without 1.5 mM hydrazine in a potential range from −0.6 V to 0.7 V. For comparison, CVs were recorded for bare GC (Figure S3), GC/MWCNT, GC/MWCNT/LS, and GC/MWCNT/LS/NPd (Figure 7). It can be seen that the background current of the bare GC electrode (Figure S3) and the electrode modified with pristine MWCNTs (Figure 7A) is significantly lower and increases when the material is further modified with LS (Figure 7B) and Pd nanoparticles (Figure 7C). This behaviour can be ascribed to the higher hydrophilicity of both MWCNT/LS and MWCNT/LS/NPd as compared to pristine MWCNTs or bare GC electrodes. At the GC and GC/MWCNT electrodes, the hydrazine oxidation process generates an anodic wave growth without a characteristic peak current. Such CV curves are due to the hindered kinetics of hydrazine oxidation on the studied electrodes.

Figure 7B shows the CV response of the GC/MWCNT/LS electrode. Its characteristics were typical for LS-modified electrodes. A representative reversible quinone/hydroquinone redox couple was observed at E^0′^ = 0.17 V. In the presence of 1.5 mM hydrazine, the oxidation signal of quinone groups recorded at 0.167 V was enhanced by hydrazine-mediated charge exchange. The well-defined sharp symmetrical peak indicates good sensitivity, selectivity, and high precision for hydrazine detection. The slight negative shift of the anodic peak is due to the presence of quinone/hydroquinone groups from LS, which can accelerate the oxidation of hydrazine [85,86]. Therefore, GC/MWCNT/LS exhibits electrocatalytic properties towards hydrazine oxidation.

Further, at the GC/MWCNT/LS/NPd electrode (Figure 7C), the oxidation peak and one reduction peak were recorded in the presence of hydrazine. The peaks’ potentials are ca. −0.09 V and −0.20 V, respectively. It was found that the oxidation peak at −0.09 V corresponds to the process of hydrazine oxidation in PB buffer electrolyte (pH = 7.4) [36,87]. Based on the results of Miao and Compton [87,88], we attributed the anodic peak to the oxidation of N_2_H_4_ in a four-electron transfer reaction, according to the following Equation (2):

(2) $$N_{2}H_{4}\overset{-4e}{\to }N_{2}+{4H}^{+}$$

Miao and Compton [81] found that hydrazine undergoes oxidation at pH 7.4 through multiple stages. One of the intermediates is the radical dication N_2_H_5_^•2+^, which is created through the first electron loss from N_2_H_5_^+^. The reduction signal of the radical dication is closely linked to the pH of the electrolyte. In the assays carried out in a PB buffer (0.05 M) with a pH of 7.4, N_2_H_5_^•2+^ remains stable, which allows the peak to be recorded at −0.20 V. A higher pH reduces the formation of the radical dication, whereas a lower pH strongly decreases the amount of protons on the electrode and produces hydrogen, masking the reduction signal of N_2_H_5_^•2+^ [87].

Summarising the juxtaposition of the three electrodes presented, we concluded that modifying the GC surface with the MWCNT/LS/NPd hybrid material gives the best results for hydrazine determination. Although both GC/MWCNT/LS and GC/MWCNT/LS/NPd show electrocatalytic properties towards the oxidation of hydrazine, the presence of NPds allows the detection of the analyte at a more negative potential, starting from −0.09 V, which is a clear advantage of the electrode over other modified electrodes proposed so far.

Next, we performed a control experiment for the MWCNT/NPd material to determine the effect of lignosulfonate on the electrocatalytic activity in hydrazine oxidation. For this purpose, NPds were deposited on the surface of pristine, unmodified MWCNTs, and the electrode was then prepared in the same way as MWCNT/LS-NPd. Cyclic voltammograms of GC/MWCNT/NPd and GC/MWCNT/LS/NPd were recorded in the absence and presence of hydrazine (Figure S4). Hydrogen sorption occurred at the GC/MWCNT/NPd electrode, indicating the presence of palladium in the material, but the addition of hydrazine caused only a slight increase in the oxidation current. None of the three characteristic peaks of hydrazine were recorded. The use of LS significantly affects the electrocatalytic properties of MWCNT/LS-NPd in the oxidation of hydrazine. The activity of MWCNT/LS-NPd is ensured by the porous and diversified structure, which facilitates the diffusion of hydrazine molecules to the active surface.

**Figure 8:**
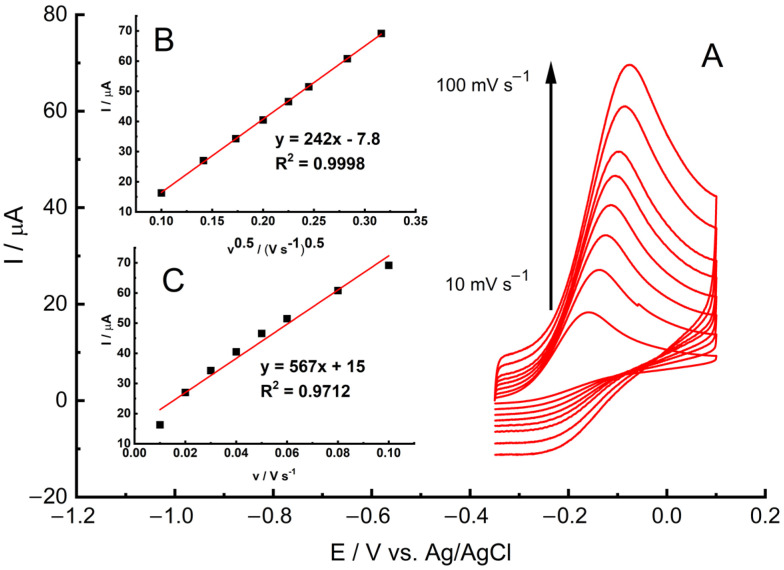
(**A**) Cyclic voltammograms registered at the GC/MWCNT/LS/NPd electrode at various scan rates from 10 to 100 mV s^−1^ in the presence of 10 mM hydrazine. (**B**) The relationship between peak current and square root of scan rate (I vs. v^1/2^). (**C**) The relationship between peak current and scan rate (I vs. v).

A study on scan rates was conducted using the GC/MWCNT/LS/NPd electrode in a PB buffer (0.05 M) with a 10 mM hydrazine solution within the range of 10 to 100 mV s^−1^. As illustrated in Figure 8, the oxidation peak current exhibited an increase with higher scan rates. Remarkably improved linearity (R^2^ = 0.9998) was achieved when plotting the catalytic current against the square root of the scan rate, denoted as I vs. ν^1/2^ (Figure 8B), in contrast to the relationship between I and v (R^2^ = 0.9712) (Figure 8C). This electrode response strongly indicates that the process is limited by diffusion. It is worth noting that this diffusion-controlled response is the preferred behaviour for the accurate application of the modified electrode in quantitative analysis.

**Figure 9:**
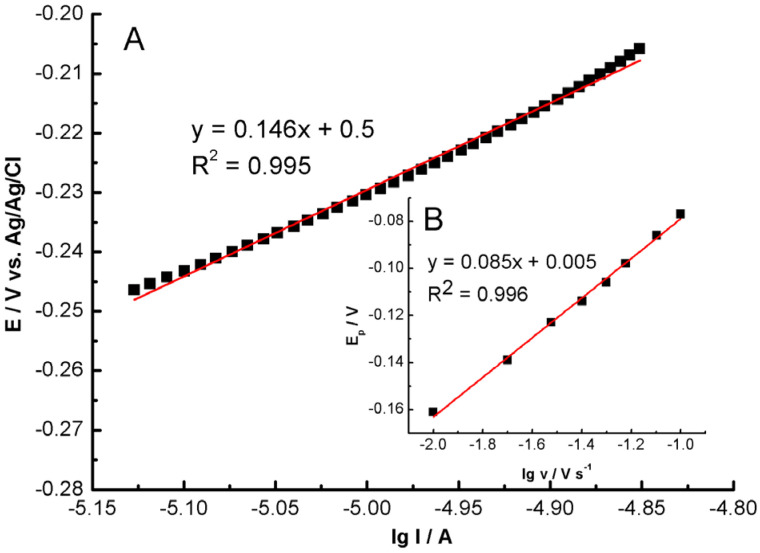
(**A**) The relationship of E vs. lg I (Tafel plot) recorded at 1 mV s^−1^ in the presence of 10 mM hydrazine. (**B**) Plot of oxidation peak potential versus logarithm of scan rate. Data collected from Figure 8A.

A Tafel plot generated from the ascending section of the current–voltage curve at a scan rate of 1 mV s^−1^ is depicted in Figure 9. The Tafel plot derived from this data exhibited a linear relationship with a slope (dlg I/dE) of 146 mV. The anodic transfer coefficient (α) was calculated to be 0.40 using Equation (3) [89]:

(3) $$\alpha =2.3{RTF}^{-1}{\left(dlgI/dE\right)}^{-1}$$

where R (8.314 J K^−1^ mol) represents the gas constant, T is the temperature (298 K), and F corresponds to the Faraday constant (96,500 C mol^−1^). The observed slope of 146 mV closely aligns with the theoretical value of 120 mV, indicative of a reaction involving a one-electron transfer in the rate-determining step, when α = 0.5 [89].

The peak potential for the catalytic oxidation of hydrazine shifts towards more positive values as the scan rate increases, implying kinetic limitations. A linear regression equation correlating potential (E_pa_/mV) and the logarithm of the scan rate (log(ν/mV s^−1^)) was established, as demonstrated in Figure 9B. The number of electron transfers in the rate-determining step can be calculated from the Tafel slope (b) using Equation (4) [90,91,92,93]:

(4) $$E_{p}=b/2\log\left(\nu \right)+constant$$

where b represents the Tafel slope. The plot of E_p_ against log (ν) exhibits a slope of 85 mV, thereby indicating a Tafel slope of 170 mV. This outcome is consistent with the results obtained from the ascending segment of the voltammogram recorded at 1 mV s^−1^, reinforcing the notion that a one-electron transfer governs the rate-determining step.

Previous research has widely supported the idea that hydrazine oxidation is a four-electron transfer reaction, with the first electron transfer considered to be the rate-determining step [90,91,92,93,94,95]. In light of these findings, a plausible mechanism for the oxidation of hydrazine can be proposed (Equations (5) and (6)):

(5) $$N_{2}H_{5}^{+}\to N_{2}H_{3}+{2H}^{+}+e^{-}\;(slow)$$

(6) $$N_{2}H_{3}+{3H}_{2}O\to N_{2}+{3H}^{+}+{3e}^{-}\;(fast)$$

The cyclic voltammetry technique was used to examine how the GC/MWCNT/LS/NPd electrode responded to varying concentrations of hydrazine (Figure S5). As the hydrazine concentration increased, there was a gradual rise in the peak current at −0.09 V. The oxidation current is linearly dependent on the hydrazine concentration in the electrolyte and has an R^2^ value of 0.998, indicating high sensitivity of the electrode to the electrocatalytic oxidation of hydrazine. However, at higher concentrations of hydrazine, the oxidised products can gradually adsorb onto the electrode surface. To precisely investigate the oxidation of hydrazine in upcoming experiments, the chronoamperometric approach was employed. This method entails using a rotating electrode which ejects the oxidised products from the electrode surface, allowing for the further reaction of analyte molecules.

**Figure 10:**
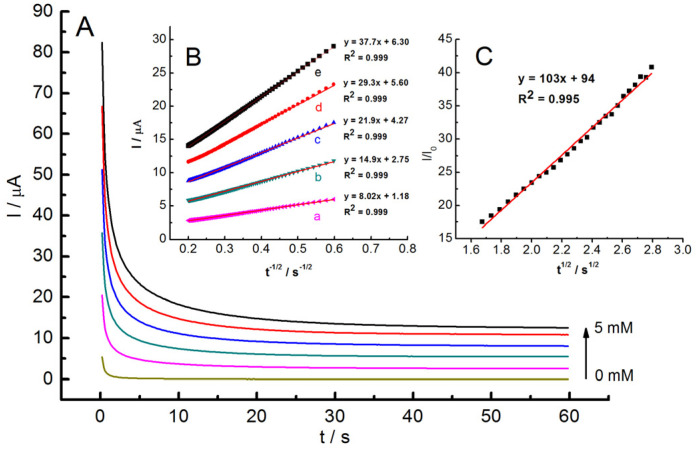
(**A**) Chronoamperometric responses recorded at the activated GC/MWCNT/LS/NPd electrode at 0.1 V at various concentrations of hydrazine from 1 to 5 mM in PB buffer, pH 7.4. (**B**) shows the plots of I vs. t^−1/2^ for the same concentrations of hydrazine. (**C**) displays the I/I_0_ vs. t^1/2^ for 5 mM hydrazine.

Next, chronoamperometric investigations were conducted to determine the diffusion coefficient (D) and electrocatalytic rate constant (k_cat_). As illustrated in Figure 10A, a series of distinct chronoamperograms were recorded at 0.1 V, both in the absence and presence of hydrazine at concentrations ranging from 1 to 5 mM. In the presence of hydrazine, the recorded currents are regulated by diffusion and can be described by the Cottrell equation (Equation (7)) [90,91,92,93,94,95]:

(7) $$I=nFAc_{0}D^{1/2}\pi^{-1/2}t^{-1/2}$$

where D and c_0_ represent the diffusion coefficient (cm^2^ s^−1^) and bulk concentration (mol cm^−3^) of hydrazine, respectively. A corresponds to the electroactive electrode area (0.069 cm^2^, estimated from the CV in the presence of [Fe(CN)_6_]^3−^ (please refer to the supporting material, Figure S6). I signifies the diffusion-controlled current of hydrazine oxidation.

The I vs. t^−1/2^ plots at varying hydrazine concentrations exhibit linearity within specific time intervals, indicating that the oxidation process conforms to the conditions outlined by the Cottrell equation (Figure 10B). The slopes of these plots were used to calculate an average value of D, which was determined to be 2.50 × 10^−7^ cm^2^ s^−1^.

Electrochemical impedance spectroscopy (EIS) measurements were conducted on the electrode surface to gain a deeper insight into the interfacial processes. Figure S7 displays customary Nyquist graphs of the unmodified GC, GC/MWCNT, and GC/MWCNT/LS/NPd with 1 mM [Fe(CN)_6_]^3−/4−^ (molar ratio of 1:1) present. The obtained data validate earlier CV findings and indicate an amplified peak differentiation with each progressive modification stage. Specifically, for GC, the value is 96 mV, for GC/MWCNT, it is 83 mV, and for GC/MWCNT/LS/NPd, the value is 76 mV (refer to Figure S6). The Nyquist diagram depicting the bare GC demonstrates a significant semicircle in the high-frequency region and a linear trend in the low-frequency zone. The high-frequency segment corresponds to the electron transfer resistance, while the linear section in the medium–low-frequency range is linked to capacitance and diffusion resistance. The EIS curves for the GC/MWCNT and GC/MWCNT/LS/NPd electrodes imply the occurrence of rapid electron conveyance and changing character to capacitive. This is connected with the highly conductive and porous character of MWCNTs. It can be deduced from both the CV (Figure S6) and EIS (Figure S7) analyses that GC/MWCNT/LS/NPd can be employed to improve the GC electrode surface with high conductivity and electroactivity [90,91,92,93,94].

Next, the chronoamperometry data were used to estimate the kcat of the oxidation of hydrazine. At halftime, the diffusion-controlled current is dominated by the rate of the electrocatalytic oxidation of hydrazine; hence, the kcat for the chemical reaction between hydrazine and the catalyst on the electrode’s surface can be determined according to Equation (8) [49]:

(8) $$I_{cat}/I_{0}=\pi^{1/2}{\left(k_{cat}c_{0}t\right)}^{1/2}$$

where I_0_ and I_cat_ are the currents recorded in the absence and in the presence of hydrazine, respectively, k_cat_ (M^−1^ s^−1^) is the second-order catalytic rate constant, c_0_ (M) is the concentration of hydrazine, and t (s) is time elapsed. The plot of I/I_0_ vs. t^1/2^ for 5 mM of hydrazine was linear (Figure 10C). From this slope, the k_cat_ was estimated to be 6.76 × 10^5^ M^−1^ s^−1^.

**Figure 11:**
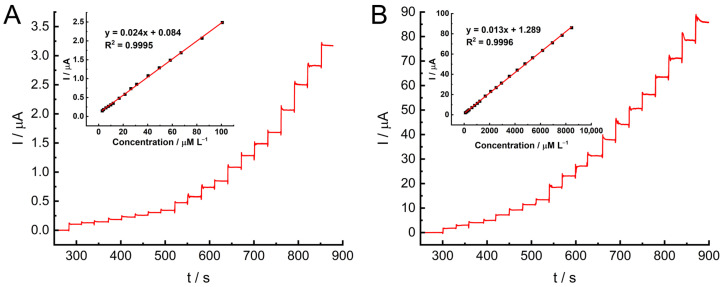
(**A**) Chronoamperometric analysis of hydrazine determination in the range of 1–100 µM of hydrazine at GC/MWCNT/LS/NPd electrode in stirring conditions (1000 rpm). Operation potential: 0.0 V. The inset is the plot of peak current versus concentration of hydrazine added. (**B**) Same as (**A**) in the range of 100–10,000 µM of hydrazine. The inset is the plot of peak current versus concentration of hydrazine added.

The chronoamperometric test was then performed at the GC electrode under optimized conditions in PB buffer at a constant stirring rate of 1000 rpm (Figure 11). To ensure the precise determination of hydrazine during its electro-oxidization process, measurements were recorded at 0.0 V. For the experiments, two hydrazine injection ranges were used, i.e., 1–100 µM (Figure 11A) and 100–10,000 µM (Figure 11B). Four injections were consecutively applied at each concentration. In both ranges, the recorded response changed at different hydrazine concentrations and was almost linearly proportional to the hydrazine concentration in the electrolyte across the entire region. For concentrations ranging from 3.00 to 100 µM L^−1^, the linear regression equation was determined as I (μA) = (0.024 ± 0.0001) C + (0.084 ± 0.0067), with an R^2^ value of 0.9995. The limit of detection (LOD) was calculated to be 0.80 µM L^−1^ using the formula LOD = (K × SD_a_)/b, where K = 3.3 and b is the slope. The limit of quantification (LOQ) was found to be 2.42 µM L^−1^ based on the equation LOQ = 3 × LOD. For the second linear range, 100–10,000 µM L^−1^, the equation was I (μA) = (0.013 ± 0.00004) C + (1.289 ± 0.18057), with R^2^ = 0.9996.

The existence of two linear ranges is presumably due to the adsorption of the hydrazine oxidation product on the electrode surface with increasing hydrazine concentration, which prevents further hydrazine diffusion [1,49,96]. Table 1 presents noteworthy outcomes for the GC/MWCNT/LS/NPd electrode in comparison to other modified electrodes utilised for hydrazine detection. The electrode presented in this study exhibits a broader linear range and a significantly lower limit of detection than that of electrodes previously reported. Furthermore, our electrode demonstrates superior electrocatalytic performance in the detection of hydrazine at a lower working potential in the PB electrolyte. Previous studies have reported hydrazine detection at potentials ranging from 0.1 V to 0.5 V [3,7,11,30,33,49,86,97,98,99,100,101]. A significant advantage of the electrode design lies in its high level of sensitivity, equal to 0.166 µA µM^−1^ cm^−2^ according to the equation sensitivity = slope/active surface area.

We performed a repeatability and reproducibility study of the modified electrode for the determination of N_2_H_4_ through the use of chronoamperometry at a constant stirring rate of 1000 rpm. The electrolyte used was PB buffer, and the working potential was 0.0 V. In order to determine the repeatability on a single GC/MWCNT/LS/NPd electrode, five equal analyte determinations were conducted, each with a concentration of 50 μM N_2_H_4_ (Figure S8). The relative standard deviation (RSD) was 3.8%, which did not significantly affect the analytical validity of hydrazine determination using the proposed electrode. The reproducibility of N_2_H_4_ determination was evaluated with five successive GC/MWCNT/LS/NPd electrodes that were prepared in an identical manner (Figure S9). The recorded currents and the calculated RSD value of 6.5% indicate that the produced electrode exhibits excellent reproducibility.

Additionally, an investigation into the stability of the MWCNT/LS/NPd hybrid material was conducted in 0.05 M PB buffer (Figure S10). A GC/MWCNT/LS/NPd electrode was prepared using a drop-casting method when the hybrid material was freshly synthesized. Subsequently, cyclic voltammetry was employed to record the electrode’s performance. The hybrid material was stored for two years in a dark glass vial filled with DMF. Then, the electrode was prepared and tested in the same way as before, recording the CV in 0.05 M PB buffer. Figure S10 displays the cyclic voltammetry (CV) results of both electrodes, which demonstrate corresponding characteristics. Analogous mechanisms of palladium oxidation/reduction and hydrogen sorption/accumulation were observed correspondingly. The electrode material remained unchanged with no indication of degradation.

The next step was to investigate the interfering effect during hydrazine detection due to the presence of common interfering biological compounds and diverse ions that may be present in tap water samples, i.e., SO_4_^2−^, NO_2_^−^, GLU, Na^+^, NO_3_^−^, and K^+^ (Figure S11). The GC/MWCNT/LS/NPd electrode was evaluated with the standard addition method, spiking 100 μM L^−1^ of hydrazine into 1.0 mM L^−1^ of each interfering substance. The recorded currents were compared with the current value recorded for 100 µM L^−1^ of pure hydrazine. Based on the results, it was found that in the presence of 10 times higher concentrations of interfering substances, the detectability of hydrazine ranges from 98.9% to 100.5%. The anti-interference properties of the developed GC/MWCNT/LS/NPd modified electrode were found to be very satisfactory for detecting and determining hydrazine.

We also carried out a standard addition test to confirm the usefulness of the proposed electrode. Measurements was carried out in a 0.1 M KCl (pH ~ 7) electrolyte at an operating potential of 0.0 V. A concentration of 100 µM L^−1^ of N_2_H_4_ was injected into the electrolyte, followed by an injection of 500 µM L^−1^ of tap water. Then, five different concentrations of hydrazine (100, 200, 300, 400, and 500 µM L^−1^) were injected into the electrolyte, and the current response was recorded. The results are summarised in Table 2. The concentration of hydrazine detected was averaged from five repeated measurements and calculated using the regression equation I (μA) = (0.013 ± 0.00004) C + (1.289 ± 0.18057). The obtained recoveries range from 99.3 ± 1% to 100.5 ± 0.6%, indicating the high accuracy of the developed electrode.

## 3. Experimental Section

### 3.1. Materials and Reagents

Multi-walled carbon nanotubes (MWCNTs) with an average diameter of 10 nm and an average length of 1.5 μm were supplied by Metrohm DropSens (Oviedo, Spain). Avantor (Gliwice, Poland) supplied monosodium and disodium phosphates for preparing phosphate buffer (PB, pH 7.4). Palladium(II) chloride ≥ 99.9% was purchased from Sigma-Aldrich (Poznań, Poland). Hydrazine (40%), perchloric acid (70%), potassium chloride, sodium hydroxide, potassium hexacyanoferrate(II) trihydrate, and potassium hexacyanoferrate(III) were purchased from Avantor (Gliwice, Poland). All chemical compounds utilised were reagent-grade and employed without additional purification. Technical lignosulfonate (LS) DP-841 (derived from softwood, Mn = 4800 Da, organic sulphur content = 6%, phenolic OH content = 2.2%) was procured from Boregaard Lignotech (Sarpsborg, Norway).

### 3.2. Apparatus

Atomic force microscopy (AFM) images were obtained using an Agilent 5500 microscope in intermittent contact mode under ambient conditions. FT-IR spectra were recorded using a Bruker FT-IR IFS 66/s. The crystal structure of the materials was analysed using X-ray diffraction (XRD, Bruker D8 Advanced, Bruker, Billerica, MA, USA). X-ray photoelectron spectroscopy (XPS) plot were acquired using a SPECS spectrometer with a monochromatic Al-Kα source, which emitted photons with an energy of 1486.71 eV (150 W), and a hemispherical analyser (PHOIBOS 150 MCD NAP), adjusted to use a pass energy of 60 eV and 20 eV for the survey and specific regions. The XPS measurements were carried out with a flood gun in an ultra-high vacuum (UHV) with a pressure below 5 × 10^−9^ mbar. The pH of the buffer solutions was determined using an Elmetron CX-505 110 pH meter with an ERH-11 electrode. Electrochemical analyses were conducted utilizing an Autolab (PGSTAT-30) electrochemical analyser (Metrohm Autolab BV, Utrecht, The Netherlands). Electrochemical impedance spectroscopy (EIS) tests were carried out at an applied potential of 0.2 V, in a time condition of 20 s, in the frequency range from 0.1 to 100,000.0 Hz, at a potential amplitude of 0.1 V. A three-electrode system, consisting of an Ag/AgCl (3 M KCl) reference electrode and a platinum wire counter electrode, was employed. The working electrode was a 2 mm diameter glassy carbon (GC) electrode from MINERAL (Sadowa, Poland).

### 3.3. Preparation of MWCNT/LS Hybrid Material

The method of synthesising MWCNT/LS material closely resembled that outlined in our previous publication [56]. LS suspensions of varying concentrations, from 0.2 to 20 mg mL^−1^, were first prepared in distilled water. Next, 20 mg of MWCNT was added to 5 mL of each LS suspension. The mixture was then sonicated for 10 min, followed by vigorous stirring for 48 h. Finally, the suspensions were centrifuged for 10 min and washed with deionised water to eliminate any loosely adsorbed LS. The purification procedure was repeated three times. MWCNT/LS hybrids were dried in an oven at 60 °C, followed by redispersion in N,N-dimethylformamide (DMF) to achieve a 1 mg mL^−1^ suspension. The purpose of this synthesis method was to determine the optimum LS loading on the surface of MWCNTs.

### 3.4. Preparation of MWCNT/LS/NPd Electrode Material

The preparation of the hybrid materials began with the gradual addition of 15 mL of 0.01 M PdCl_2_ to 20 mg of desiccated MWCNT/LS materials with constant stirring. A magnetic stirrer was used to stir the mixtures for a period of 1 h following the addition. The resultant suspensions underwent centrifugation at 6000 rpm for 10 min, subsequent decantation, and were then rinsed with distilled water. The cleansing procedure was repeated three times. The materials obtained were subjected to complete draining after being placed in an oven set at 60 °C. Subsequently, the resulting powders were re-dispersed in DMF (1 mg mL^−1^).

### 3.5. Preparation of GC/MWCNT, GC/MWCNT/LS, and GC/MWCNT/LS/NPd Modified Electrodes

Prior to every electrochemical measurement, the GC electrode underwent polishing using an aqueous Al_2_O_3_ suspension (50 nm average diameter, Buehler, Leinfelden-Echterdingen, Germany) on a polishing cloth. Following this, it was rinsed in a 1:1 *v*/*v* water/acetone ultrasonic bath for 10 min, aiming to eliminate any contamination.

To modify the GC electrode, pristine MWCNT, MWCNT/LS, and MWCNT/LS/NPd materials were drop-casted on 2 μL of each suspension (1 mg mL^−1^ in DMF) onto the cleaned GC surface. The electrode was placed in an oven to evaporate the solvent. Afterwards, all electrodes underwent electrochemical testing following their immersion into the electrolyte.

## 4. Conclusions

This work proposes a successfully developed hybrid material based on multi-walled carbon nanotubes coated with lignosulfonate and decorated with palladium nanostructures. The obtained composite showed excellent catalytic activity and a fast response to hydrazine. It was successfully used as an electrode material for the electro-oxidation of hydrazine and can be applied in the future to develop a new amperometric sensor for the determination of hydrazine at a low potential of 0.0 V. GC/MWCNT/LS/NPd showed remarkable activity and sensitivity, equal to 0.166 µA µM^−1^ cm^−2^. A wide linear range (from 3 to 100 µM L^−1^ and from 100 to 10,000 µM L^−1^) and a low detection limit (0.80 µM L^−1^) are undoubtedly the major advantages of the presented electrode. In addition, negligible interferences were observed in the presence of biological compounds and various ions. The proposed electrode also showed excellent electrocatalytic properties for the detection of hydrazine in tap water samples. It has been demonstrated that MWCNT/LS/NPd is an easy and cheap material to prepare and is an excellent alternative for the development of electrocatalytically active materials for hydrazine detection.

## Figures and Tables

**Table 1:** Comparison of different modified electrodes for hydrazine detection.

Electrode Material	Linear Range [μM]	LOD [μM]	Reference
GC/MWCNT/LS/NPd	3–100, 100–10,000	0.80	This work
Pd-PEDOT@CM/GNP/GCE	1–100, 100–5000	0.28	[49]
Pd/MWNTs/GCE	2.5–700	1.0	[11]
Pd/CNF/GCE	10–4000	2.9	[97]
Pd/CB/GCE	5–500	8.8	[7]
Pd-GCM/MWCNT/GCE	2–500	0.6	[43]
PEDOT/Pd	0.5–30	0.8	[46]
PdNPs wrapped with guar gum-grafted-PAM-silica material/GCE	50–600	4.1	[102]
AuNP-GPE	25–1000	3.07	[98]
PVP-AgNCs/GCE	5–460	1.1	[30]
Co_3_O_4_/GCE	50–1088	3.7	[99]
Au@Pd/CB-DHP/GCE	2.5–88	1.77	[3]
NiCo_2_S_4_/GCE	1.7–7800	0.6	[100]
CoS_2_/IL-Gr/GCE	5–100, 100–400	0.39	[101]
GCE/PPy/LS	2–75	1.65	[86]
GCE/PEDOT/LS	15–290, 380–2300	9.80	[86]

**Table 2:** Detection of hydrazine in tap water samples using GC/MWCNT/LS/NPd.

Water Samples	Added Hydrazine [μM]	Found [μM]	Recovery [%]
S1	0	N.D.	-
S2	100	99.6 ± 0.3	99.6 ± 0.3
S3	200	201.2 ± 1.2	100.6 ± 0.6
S4	300	300.4 ± 2.7	100.1 ± 1.0
S5	400	397.3 ± 3.1	99.3 ± 1.0
S6	500	492 ± 3.2	98.6 ± 1.0

## Data Availability

Data sharing is not applicable to this article.

## Supplementary Materials

The following supporting information can be downloaded at: https://www.mdpi.com/article/10.3390/molecules28207076/s1.

## Sample Availability

Not available.
