# Supplementary material for: Lignosulfonate-Assisted In Situ Deposition of Palladium Nanoparticles on Carbon Nanotubes for the Electrocatalytic Sensing of Hydrazine
Source: Molecules. 2023 Oct 13;28(20):7076. doi: 10.3390/molecules28207076 (PMC10609262; doi:10.3390/molecules28207076)
Supplement: Supplementary file 1 [file molecules-28-07076-s001.zip › molecules-2607077-supplementary.pdf]

Lignosulfonate-assisted in situ deposition of palladium nanoparticles on carbon nanotubes for the electrocatalytic sensing of hydrazine

Patrycja Płócienniczak[1], Tomasz Rębiś[2]*, Amanda Leda[2], Grzegorz Milczarek[2]*

[1]*Faculty of Chemistry, Adam Mickiewicz University, Uniwersytetu Poznańskiego 8, 61-614 Poznań, Poland*

[2]*Institute of Chemistry and Technical Electrochemistry, Poznan University of Technology, Berdychowo 4, 60-965 Poznań, Poland*

Supporting Material

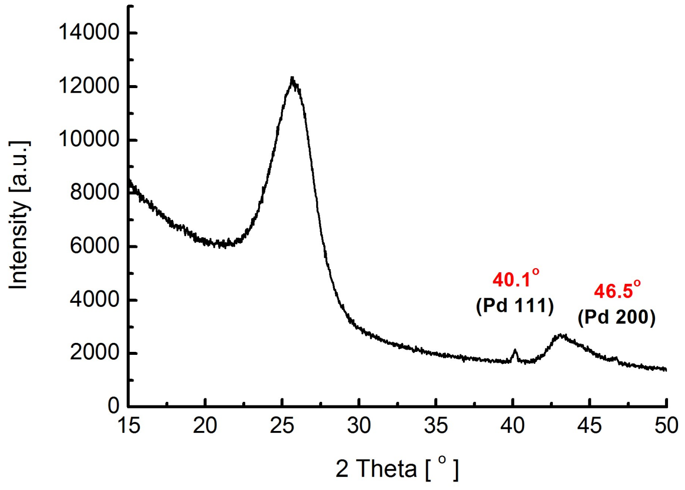

Fig. S 1. XRD profile of MWCNT/LS/NPd hybrid material with the indication of characteristic palladium peaks.

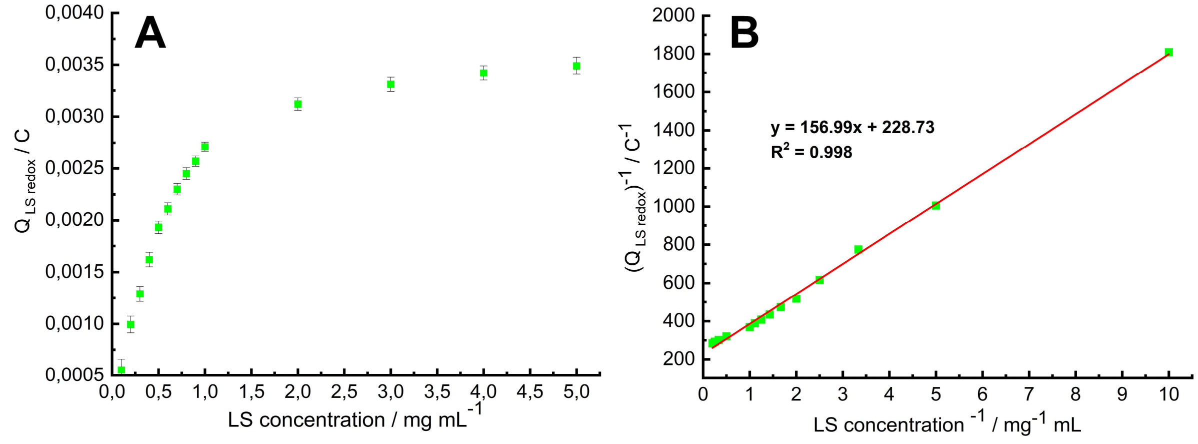

Fig. S 2. (A) LS redox peak charge measured for different MWCNT/LS samples as a function of the LS concentration used for synthesis (concentration range 0.1 to 20.0 mg mL^{-1}). (B) Relationship between the inverse of the redox peak charge and the inverse of the LS concentration.

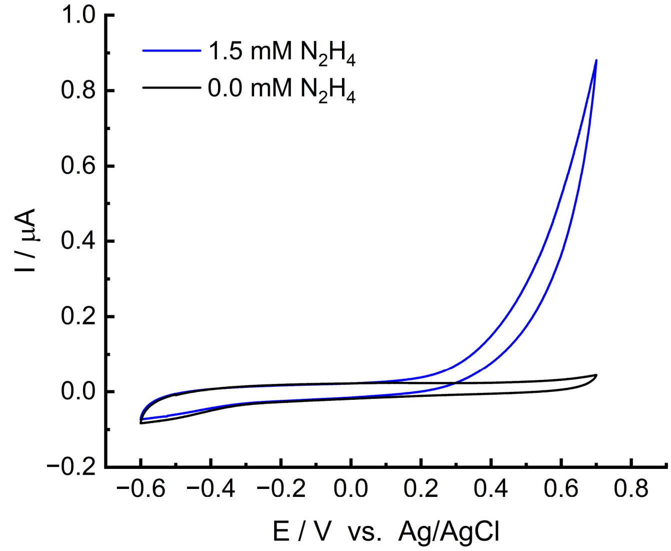

Fig. S 3. CV of bare GC electrode recorded in 0.05 M PB buffer in the absence and presence of 1.5 mM N_2H_4. Scan rate: 10 mV s^{-1}.

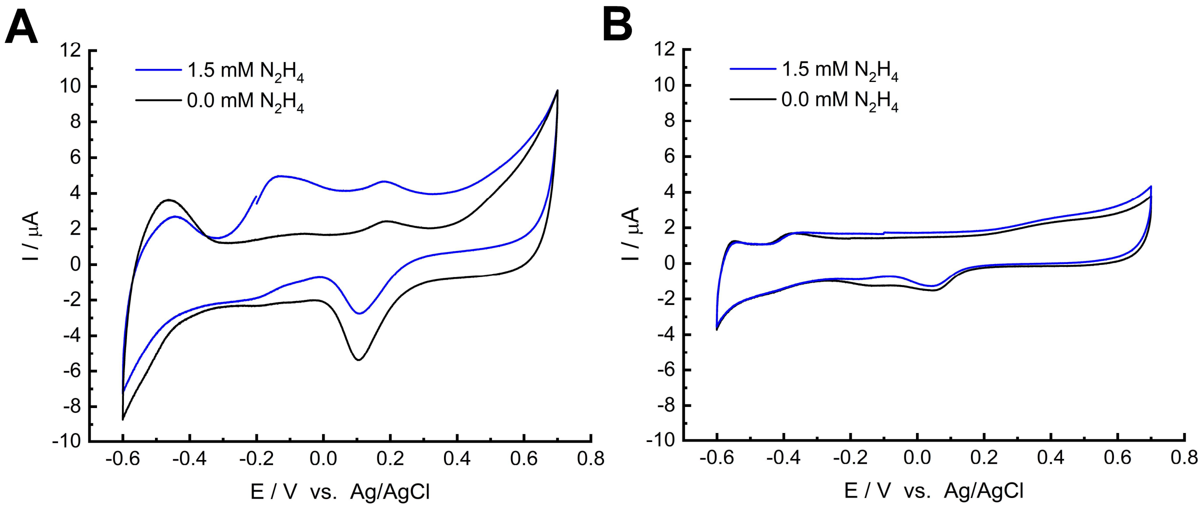

Fig. S 4. Cyclic voltammograms registered in 0.05 M PB buffer solution before and after the addition of 1.5 mM N_2H_4 at (A) GC/MWCNT/LS/NPd and (B) GC/MWCNT/NPd. Scan rate 10 mV s^{-1}.

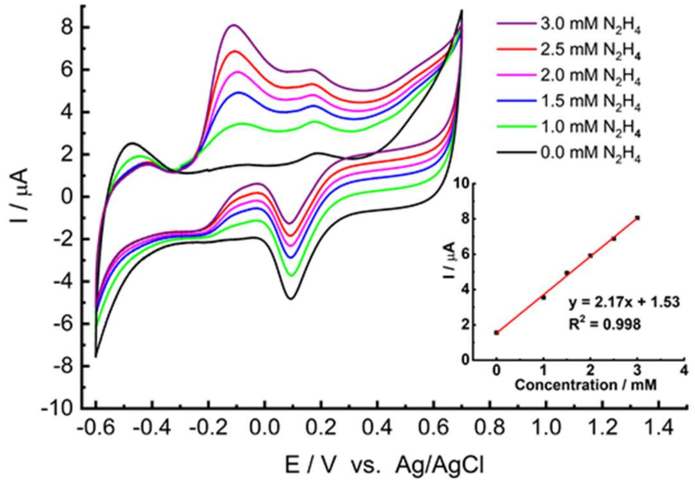

Fig. S 5. CVs of GC/MWCNT/LS/NPd, recorded for subsequent hydrazine additions in PB buffer solution (0.05 M) at a scan rate of 10 mV s^{-1}. The inset is the plot of peak current versus concentration of hydrazine at -0.09 V.

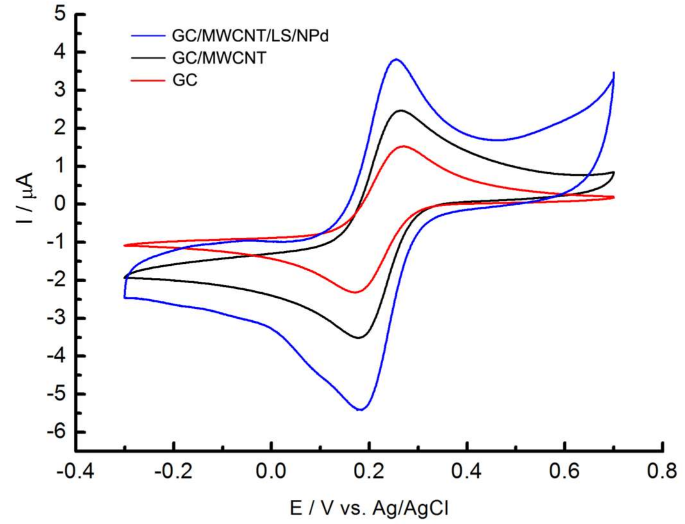

Fig. S 6. Cyclic voltammograms recorded at bare GC, GC/MWCNT and GC/MWCNT/LS/NPd in the presence of 1.0 mM $[Fe(CN)_6]^{3-}$ at 10 mV s^{-1}. Supporting electrolyte is PB buffer, pH 7.4.

According to Randles-Sevcik:

$I_p = 2.69 \times 10^5 n^{3/2} A C D^{1/2} v^{1/2}$

A (GC) = 0.03 cm^2
A (GC/MWCNT) = 0.046 cm^2
A (GC/MWCNT/LS/NPd) = 0.069 cm^2

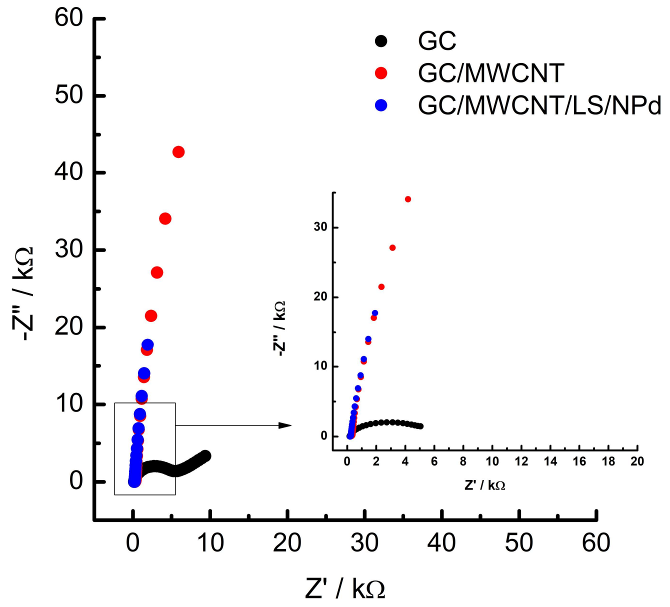

Fig. S 7. Nyquist diagrams of electrodes in PB solution (pH 7.4) containing 1 mM $[Fe(CN)_6]^{3-/4-}$.

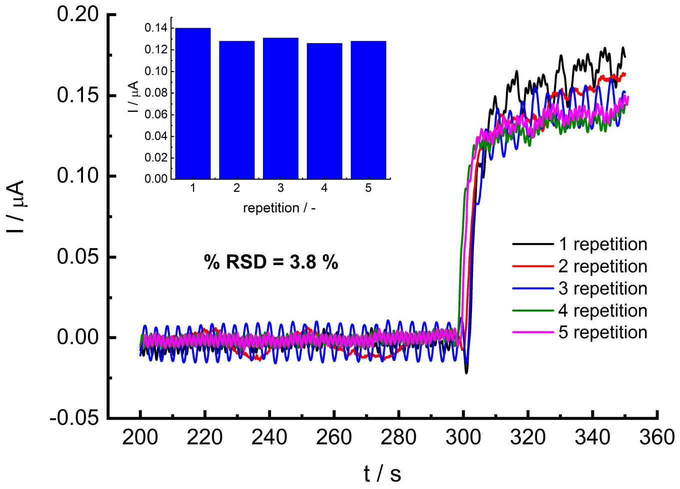

Fig. S 8. Repeatability of GC/MWCNT/LS/NPd for the detection of hydrazine (50 μM) in PB buffer. Operation potential 0.0 V.

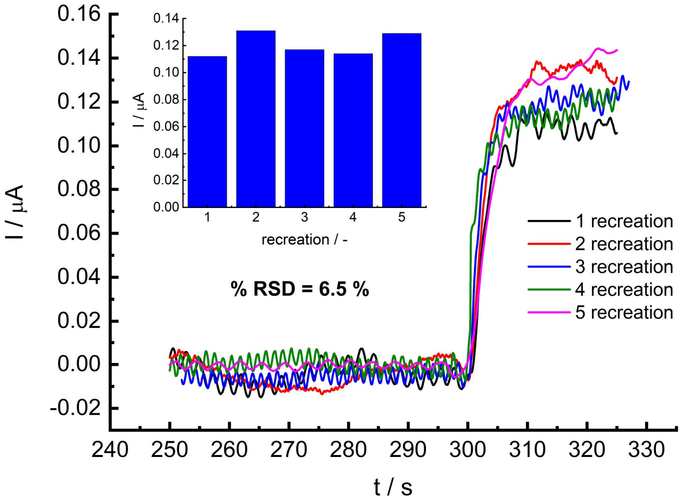

Fig. S 9. Reproducibility of GC/MWCNT/LS/NPd for the detection of hydrazine (50 µM) in PB buffer. Operation potential 0.0 V.

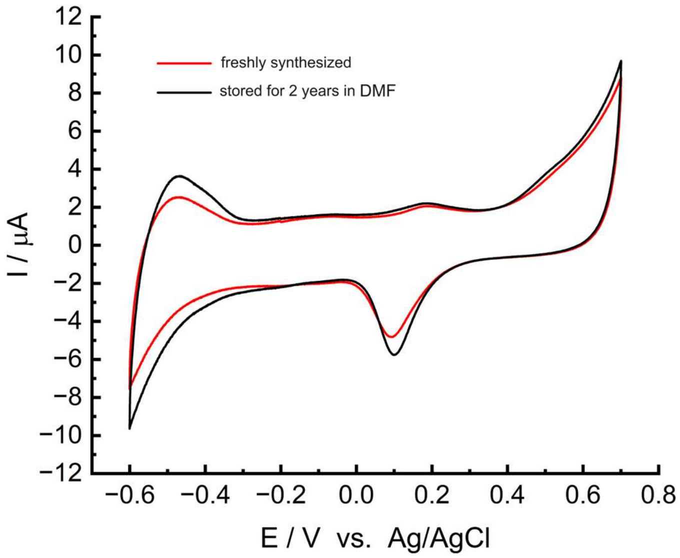

Fig. S 10. Cyclic voltammograms were recorded in 0.05 M PB buffer solution on identical GC/MWCNT/LS/NPd electrodes. The first electrode incorporated the freshly synthesized hybrid material, while the second one contained the hybrid material that had been stored in DMF for 2 years. Scan rate 10 mV s^{-1}.

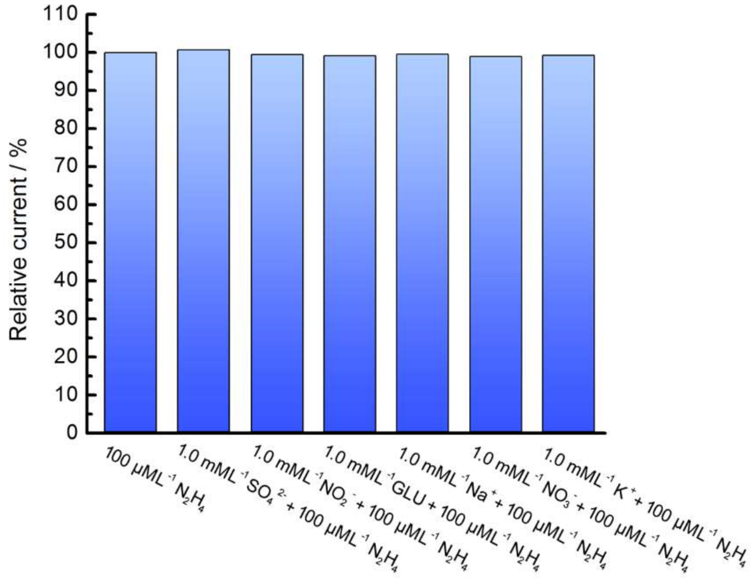

Fig. S 11. Interfering effect during hydrazine detection at GC/MWCNT/LS/NPd.
